# Expectations of individuals with neurological conditions from rehabilitation: A mixed-method study of needs

**DOI:** 10.4102/sajp.v77i1.1498

**Published:** 2021-01-15

**Authors:** Olubukola A. Olaleye, Desmond A. Zaki, Talhatu K. Hamzat

**Affiliations:** 1 Department of Physiotherapy, Faculty of Clinical Sciences, University of Ibadan, Ibadan, Nigeria; 2 Department of Physiotherapy, Benue State University Teaching Hospital, Markurdi, Nigeria

**Keywords:** rehabilitation, needs assessment, expectations, disability, stroke, spinal cord injury, traumatic brain injury

## Abstract

**Background:**

Knowledge of the specific expectations of patients with neurological conditions (NCs) from rehabilitation helps in setting attainable goals. Such expectations may vary from situation to situation. There are no studies investigating rehabilitation expectations amongst individuals with NCs in Nigeria.

**Objectives:**

The aim of our study was to explore the rehabilitation expectations of individuals with NCs.

**Method:**

This convergent mixed-methods study comprised a cross-sectional survey of 105 individuals with NCs and two sessions of Focus Group Discussions (FGDS) amongst eight individuals with NCs. The modified Needs Assessment Questionnaire was used to assess rehabilitation needs as a proxy for rehabilitation expectations, whilst disability was assessed using the World Health Organization Disability Assessment Schedule 2.0. Quantitative data were summarised using descriptive statistics and analysed using inferential statistics at *p* < 0.05. Thematic analysis was conducted on the qualitative data.

**Results:**

Sixty-one (58.1%) stroke survivors, 33 (31.4%) individuals with spinal cord injury (SCI) and 11 (10.5%) with traumatic brain injury (TBI) aged 46.48 ± 15.91 were surveyed. The need for social/recreational activity was the most expressed need (100%) amongst the participants. Mobility was reported as an important need constituting a barrier to enjoying life by 93 (88.6%) participants. Individuals with SCI expressed the greatest needs compared with the other two groups. Needs were significantly correlated with severity of disability (*p* < 0.05). Four overarching themes (physical health, financial, healthcare services/rehabilitation and emotional/social) representing major areas of needs emerged from the FGD data.

**Conclusion:**

Individuals with NCs in Nigeria have specified expectations of rehabilitation. Disability was a major driver of these expectations, irrespective of NC subtype.

**Clinical implications:**

Rehabilitation programmes for individuals with NCs should target expressed needs or expectations of each patient cohort and minimise disabilities associated with these conditions.

## Introduction

Neurological conditions (NCs) are a frequent cause of morbidity and disability, which constitute about 12% of total deaths globally (WHO 2006). The potential impact of NCs ranges from physical, motor, sensory, cognitive and communication impairments to psychosocial ones (Mohamed 2014). The impact of NCs on the overall well-being of individuals can be challenging, particularly in low- to middle-income countries where rehabilitation services and assistive technology are limited (WHO & World Bank 2011). Rehabilitation plays a key role in the management of people with NCs. It also minimises disability and improves the quality of life of people with acute and chronic NCs (Macdonell & Dewey 2001). The goal of rehabilitation services is to achieve and maintain optimal functioning in the short and long term (Rauch et al. 2011).

Traditionally, rehabilitation services for individuals with NCs have reflected the needs and priorities identified by healthcare professionals. It has, however, been suggested that rehabilitation needs and the resources required to meet them are perceived differently by individuals requiring them and the healthcare professionals (Kersten et al. 2000). International best practices require an assessment of rehabilitation needs from a patient or client’s perspective (Kristensen et al. 2016). Measuring patients’ own perceptions of their rehabilitation needs are essential in identifying, prioritising and determining goals and priorities in rehabilitation and in promoting independence in the community (Harris & Eng 2004). It helps in tailoring rehabilitation services to the expressed needs of the affected population (Rauch et al. 2011). Also, organising rehabilitation around patients’ expressed needs encourages their involvement in rehabilitation, enhances confidence in clinicians and improves satisfaction with care (Kennedy, Smithson & Blakey 2012; Whalley Hammell 2007). However, rehabilitation needs of patients with NCs are rarely assessed (Lynch et al. 2015).

Although some authors have examined the needs of individuals with some specific NCs, most of the available studies have been conducted in high-income countries. DePaul, Moreland and Dehueck (2013) report needs and barriers related to motor control, walking, stairs, fatigue, prevention of falls and access to physiotherapy services amongst patients with stroke in Canada. Needs related to mobility, arm and hand rehabilitation, general exercise, fall prevention and access to physiotherapy were also reported in earlier studies, all involving stroke survivors (Moreland et al. 2009; Pierce, Gordon & Steiner 2004; Talbot et al. 2004; Vincent et al. 2007). Van Loo et al. (2010) reported unmet information and psychological needs in individuals with long-term spinal cord injury (SCI) in the Netherlands. Beauregard et al. (2012) also reported high unmet emotional, psychological and employment needs amongst community-living individuals with SCI in Quebec. Fuentes et al. (2018) found unmet mental health and educational needs amongst children with traumatic brain injury (TBI) in the United States, whilst Jourdan et al. (2018) reported a high level of unmet need for information amongst adults with TBI in Paris.

Considering the differential impact of socio-economic status and culture on the level of disabilities resulting from NCs, it is possible that the rehabilitation needs of people with NCs in Nigeria will differ from those of their counterparts from other countries. There is no readily available information on the rehabilitation needs of this group of patients in our environment.

There have also been reported variations in rehabilitation needs of people with disabilities based on the primary condition causing the disability (Krahn, Walker & Correa-De-Araujo 2015). There appears to be a dearth of studies comparing rehabilitation needs across NCs. Previous studies have used either survey instruments alone or qualitative studies alone in order to investigate different aspects of rehabilitation needs of individuals with NCs. A mixed-method study assessed sexual education needs only amongst people with SCI (New et al. 2016). Using a mixed-methods design produced an extensive description of rehabilitation needs and expectations from the patients’ viewpoint. Acknowledging the expressed rehabilitation needs of patients and incorporating the same into treatment plans not only increases trust and satisfaction with care (Lateef 2011) but could also guide the development and implementation of appropriate and pragmatic rehabilitation services to address such needs. Thus, the objectives of our study were to investigate the rehabilitation needs of adults with selected NCs (stroke, TBI and SCI) in Ibadan, Nigeria, and to investigate the relationship between needs and severity of disability.

## Methods

**FIGURE 1:**
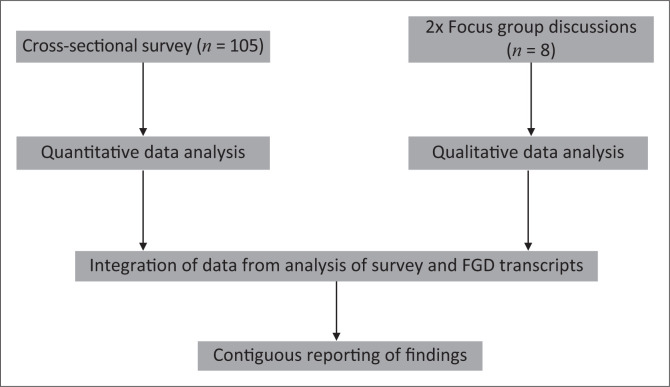
Flow chart showing participants and research procedure.

Our convergent mixed-methods study included a cross-sectional survey and Focus Group Discussions (FGDs) (Figure 1). Mixed-methods design is ideal for capturing patients’ experiences and for an in-depth understanding of a phenomenon (Morgan 2007). It is, therefore, well suited for exploring patients’ expectation of rehabilitation. The study is reported in line with the Good Reporting of A Mixed-Methods Study (GRAMMS) guideline (Appendix 1).

Participants for the cross-sectional survey were adults with stroke (61), TBI (11) and SCI (33) attending the physiotherapy clinics of three government-owned hospitals in Ibadan, Nigeria. These three NCs (Stroke, TBI and SCI) were selected because they are sudden onset NCs with significant life-altering impacts, and the main mode of management of these conditions is rehabilitation. Purposive and proportional sampling techniques were used to recruit participants for the survey. Adults diagnosed with more than one NC or those with other concomitant illnesses, which can influence or amplify their needs, were ineligible to participate in our study.

The population of patients with NCs receiving physiotherapy in the selected hospitals during the study period was determined from the available records at the hospitals as 142 (98 stroke survivors, 33 individuals with SCI and 11 individuals with TBI). The sample size for the study was determined using the formula (Yamane 1967):

$$n=\frac{N}{1+N{\left(\text{e}\right)}^{2}};N=142\text{ and }e=0.05,$$ [Eqn 1]

n=105. [Eqn 2]

Proportional sampling was subsequently used to determine the number of participants and patient groups to be recruited from each of the three hospitals (a tertiary and two general hospitals) in Ibadan. These hospitals were selected because they constitute the government-owned referral hospitals for managing patients with NCs in Ibadan, Nigeria. A total of 74 individuals with NCs comprising 37 stroke survivors, 27 individuals with SCI and 10 individuals with TBI were recruited from the University College Hospital, Ibadan (tertiary healthcare facility), 26 participants made up of 20 stroke survivors, five individuals with SCI and one individual with TBI were recruited from Ring-road State Hospital, Ibadan, whilst five participants comprising four stroke survivors and one individual with SCI were recruited from Adeoyo Maternity Teaching Hospital, Yemetu, Ibadan.

### The modified Needs Assessment Questionnaire

The NAQ was initially developed to assess rehabilitation needs of stroke survivors by Moreland et al. (2009). The original NAQ is a 160-item measure covering 13 domains. Twelve of the domains enquire about needs as experienced by the patients, whilst one domain is on barriers to enjoying life. The magnitude of each need and barrier is scored on a five-point scale from 0 = not a need to 4 indicating ‘[*a*] very large need’. The test-retest reliability of the original NAQ has been reported in the literature (DePaul et al. 2013; Moreland et al. 2009). Written permission was obtained from the developers to modify the NAQ for use amongst patients with TBI and SCI and to make changes to wording for use in a Nigerian context.

A panel of experts comprising four physiotherapists with a minimum of 15 years of experience, spanning clinical, academic and research settings, assessed the level of relevance of the items to construct the questionnaire. Experts were sourced based on their professional roles, publications and research activities. Two of the physiotherapists had expertise in outcomes development. The original version of the NAQ was given to the experts with an assessment checklist. An expert panel meeting was held to enable the panel to reach consensus on the relevance of each item and coverage of the NAQ. After the expert panel meeting, the following modifications and additions were made to the original NAQ:

Items 28 (…to do yard work), 52 (…reliable and convenient wheelchair transportation, e.g., DARTS, Handi Van, Wheel Trans), 57 (…to consider moving to a retirement or nursing home, 62 (help to start volunteer activities in the community) and 135 (…to be able to deduct my nonmedical expenses on my taxes…) were removed because of lack of relevance to a Nigerian context. The word tub (item 4) was changed to bath tub; transit (item 51) was changed to transport; domain F – needs related to volunteering or working was changed to needs related to employment or working; volunteer activities was removed from item 69; and lack of stroke therapy services close to my home (item 160) was changed to lack of rehabilitation services near my home. The entire ‘domain J’ on needs related to physical symptoms of stroke was removed from the questionnaire. The term stroke was changed to NC in items 82,83, 88–90, 136 and 147 of the original NAQ. The modified NAQ (mNAQ) used for data collection in our study comprised 141 items in 12 domains. The mNAQ was pre-tested and content validated amongst 25 patients with NCs (10 with stroke, 11 with SCI and 4 with BI). Feedback from the participants revealed the clarity of items. Cronbach’s alpha for the mNAQ was 0.98.

### World Health Organization Disability Assessment Schedule-2, 36-item version

The level of disability amongst participants was assessed using the World Health Organization Disability determining disability levels and profiles (Üstün et al. 2010). It consists of 36 items in six domains measuring cognition, mobility, self-care, getting along with people, life activities and participation. Each item is scored on a 5-point Likert scale from 1 signifying no disability to 5 indicating extreme disability. The simple scoring method, which entailed summing up the scores assigned to each item on the schedule, was used for computing the summary scores in our study. The WHODAS 2.0 has a test-retest reliability with an overall intra-class coefficient (ICC) of 0.98 and a Cronbach’s alpha of 0.98 (WHO 2010). It has correlations of between 0.45 and 0.65 with the London Handicap scale, Functional Independence Measure and the Medical Outcomes Study 36-item Short Form Health Survey, and has also been reported to be sensitive to changes with an effect size of 0.46–1.38 (WHO 2010).

### Procedure

Quantitative and qualitative data were collected concurrently.

#### Survey of expectations/needs of people with neurological condition

A detailed explanation of the procedure and relevance of the study was given to all eligible participants. They were informed that participation in the study was entirely voluntary. Participants who expressed interest were requested to sign/thumbprint the informed consent forms attached to the questionnaires. The mNAQ and WHODAS 2.0 used for the cross-sectional survey were interviewer-administered to all participants by one of the authors (DAZ) after consent was obtained. Survey data were collected from May to August 2017.

#### Exploration of expectations/needs of people with neurological conditions using focus group discussions

The needs of people with NCs were explored during FGDs. Two sessions of FGDs were held with eight patients with NCs (four stroke survivors, three patients with SCI and one patient with TBI). Participants were purposively selected from participants in the cross-sectional survey based on their ability to communicate freely and spontaneously in both English and Yoruba. All participants for the FGDs were recruited from the same healthcare facility to minimise expectations related to variation in healthcare service provision. A focus interview guide (Appendix 2) was developed based on a review of previous studies on rehabilitation needs of patients with stroke (DePaul et al. 2013; Moreland et al. 2009). The guide was reviewed by the same expert panel that evaluated the mNAQ. The FGDs were moderated by a senior lecturer with a PhD in cardio-pulmonary physiotherapy who had experience in conducting qualitative studies. The moderator had no direct link with participants who were patients with NCs. Participants in the survey were invited to volunteer for participation in the FGDs by DAZ who was on a clinical posting in the tertiary hospital as a postgraduate student. FGDs were held twice, 1 week apart, in order to ensure data saturation. The time taken for each discussion session was about 60 min in length. Discussions were audio-taped, and field notes were also taken.

### Data analysis

Quantitative data were cleaned and analysed using Statistical Package for the Social Sciences (SPSS) version 20.0. As questionnaires were interviewer-administered, there were no missing data. Descriptive statistics were used to summarise socio-demographic characteristics of participants and prevalence of rehabilitation needs derived from the mNAQ. A Spearman’s rank correlation was used to examine the relationship between rehabilitation needs and each of age and disability. A Kruskal-Wallis test was used to find the differences in rehabilitation needs across the level of education and NCs. A Mann-Whitney U test was used to find difference in rehabilitation needs between male and female participants. The level of significance was set at 0.05.

Data from the qualitative study were transcribed verbatim by a professional transcriptionist. A deductive approach was employed for the thematic analysis of transcripts. An independent data analyst who is versed in qualitative data analysis performed the initial coding of the raw transcripts. Transcripts and coding were compared and validated by two of the authors (OAO and DAZ) independently using a constant comparative technique. Themes were identified based on the frequency of occurrence in transcripts and relevance to the objectives of our study. Four main themes indicating areas of perceived rehabilitation needs emerged from the thematic analysis data and were agreed upon by the research team.

### Ethical consideration

Ethical approval was obtained from the appropriate Institutional Health Research Ethics Committee. Informed consents were obtained, and other data privacy procedures were duly followed. Socio-demographic information was elicited from participants and documented using a data capturing form. Participants were then requested to complete two patient-reported outcome measures – the modified Needs Assessment Questionnaire and the WHODAS 2.0. Ethical clearance number: UI/EC/17/0053.

## Results

Quantitative and qualitative data were merged to produce a rich and more complete description of the expectations of patients with NCs from rehabilitation using a contiguous approach (Figure 1).

### Results from the survey of expectations/needs of people with neurological conditions

A total of 105 individuals comprising 61 (58.1%) stroke survivors, 33 (31.4%) individuals with SCI and 11 (10.5%) individuals with TBI participated in the cross-sectional survey. Participants’ age ranged from 18 to 76 years with a mean of 46.48 ± 15.91. The mean time because the onset of NC was 21.03 ± 25.65 months. Socio-demographic variables of the participants are presented in Table 1.

**TABLE 1:** Socio-demographic and clinical characteristics of participants (*n* = 105).

Variables	*N*	%
**Gender**		
Male	62	59.0
Female	43	41.0
**Marital status**		
Single	32	30.5
Married	54	51.4
Divorced or separated	3	2.9
**Age ranges (years)**		
≤ 30	21	20.0
31–40	24	22.9
41–50	10	9.5
51–60	23	21.9
> 60	27	25.7
**Neurological condition**		
TBI	11	10.5
SCI	33	31.4
Stroke	61	58.1
**Level of education**		
Primary	4	3.8
Secondary	28	26.7
Tertiary	73	69.5

SCI, spinal cord injury; TBI, traumatic brain injury.

Primary education: 6 years of schooling.

Secondary education: 12 years of schooling.

Tertiary education: ≥ 16 years of schooling.

All participants expressed needs and barriers in all the domains of the mNAQ. The need for social and recreational activity was the mostly reported need by all participants, whilst the least prevalent need was employment/working need. Almost all the participants (100; 95.2%) perceived barriers to enjoying life to its fullest as a major need. The prevalence of expressed needs is presented in Table 2. The five most prevalent needs amongst participants with TBI were social/recreational need (100.0%); rehabilitation/medical need (100.0%), support in the community (100.0%), feeling/memory/emotional need (100.0%) and barriers to enjoying life (100.0%). Participants with SCI expressed the greatest needs in social/recreational need (100.0%), rehabilitation/medical need (100.0%), barriers to enjoying life (97.0%), mobility need (93.9%) and support in the community (93.9%). The most prevalent needs amongst stroke survivors include social/recreational need (100.0%), support in the community (100.0%); rehabilitation/medical need (96.7%), feelings/memory/emotional need (96.7%) and service in community and home needs (95.1%). Individuals with SCI reported significantly highest needs in mobility (< 0.01), rehabilitation and medical (*p* = 0.04), social and recreational activity (*p* = 0.03), financial and government assistance (*p* < 0.01), and barriers to enjoying life domains (*p* < 0.01) across NCs (Table 3).

**TABLE 2:** Frequency of expressed needs across neurological conditions as scored by modified Needs Assessment Questionnaire (*n* = 105).

Domains of mNAQ	TBI (*n* = 11)		SCI (*n* = 33)		Stroke (*n* = 61)		Total (*n* = 105)	
	*n*	%	*n*	%	*n*	%	*n*	%
Mobility	9	81.8	31	93.9	53	86.9	93	88.6
Self-care	10	90.9	27	81.8	53	86.9	90	85.7
Home care	10	90.9	30	90.9	56	91.8	96	91.4
Communication	9	81.8	28	84.8	45	73.8	82	78.1
Service in home and community	9	81.8	30	90.9	58	95.1	97	92.4
Employment/working	8	72.7	21	63.6	49	80.3	78	74.3
Rehabilitation/medical	11	100	33	100	59	96.7	103	98.1
Support in community	11	100	31	93.9	61	100	103	98.1
Feelings, memory and emotions	11	100	30	90.9	59	96.7	100	95.2
Social/recreational	11	100	33	100	61	100	105	100
Financial/government assistance	8	72.7	30	90.9	50	82	88	83.8
Barriers to enjoying life	11	100	32	97.0	57	93.4	100	95.2

mNAQ, modified Needs Assessment Questionnaire; SCI, spinal cord injury; TBI, traumatic brain injury.

**TABLE 3:** Differences in rehabilitation needs (as scored by modified Needs Assessment Questionnaire) across neurological conditions (***n*** = 105).

Domains of NAQ	TBI (mean rank)	SCI (mean rank)	Stroke (mean rank)	*χ*^2^	*p*
Mobility	44.14	67.47	46.77	10.98	0.01*
Self-care	67.14	53.55	50.16	2.95	0.23
Home care	61.32	53.12	51.43	0.98	0.61
Communication	65.09	53.32	50.65	2.13	0.34
Service in home and community	59.45	53.62	51.50	0.66	0.72
Employment/working	50.65	42.73	47.88	0.98	0.61
Rehabilitation/medical	61.32	62.11	46.57	6.61	0.04*
Other support in community	55.32	62.62	47.38	5.45	0.07
Feelings, memory and emotions	56.64	61.53	47.73	4.58	0.10
Social and recreational	58.32	63.39	46.42	7.06	0.03*
Financial/government assistance	58.00	68.92	43.48	15.42	0.01*
Barriers to enjoying life	58.17	63.30	41.83	12.09	0.01*

* , Significant difference at *p* < 0.05.

NAQ, needs assessment questionnaire; SCI, spinal cord injury; TBI, traumatic brain injury.

Significant differences in rehabilitation and medical need (*p* = 0.04), feelings, memory and emotional needs (*p* = 0.01), and financial and government assistance needs (*p* = 0.03) were found between male and female participants, with females expressing more needs than males. Participants with secondary education expressed the most needs in mobility (*p* < 0.01), self-care (*p* < 0.01), communication (*p* = 0.02), service at home and community (*p* < 0.01), and support in the community domains across levels of education. Although a negative correlation was found between age and 10 out of the 12 domains of mNAQ, only two domains (rehabilitation and medical needs and barriers to enjoying life) significantly correlated with age (*p* = 0.01).

Participants with SCI reported significantly worst disability in the mobility and participation domains of the WHODAS 2.0. Cognitive disability was significantly worst in patients with TBI and the best amongst participants with SCI (Table 4). There were significant linear relationships amongst almost all the domains of the WHODAS 2.0 and the domains of the mNAQ (Table 5).

**TABLE 4:** Differences in severity of disability (as scored by World Health Organization disability assessment schedule 2.0) across neurological conditions (*n* = 105).

Domains	TBI (mean rank)	SCI (mean rank)	Stroke (mean rank)	*χ*^2^	*p*
Cognition	65.68	46.03	54.48	7.23	0.03*
Mobility	49.41	72.05	43.34	19.52	0.01*
Self-care	61.50	54.74	50.52	1.38	0.50
Getting along with people	49.18	57.68	51.16	1.46	0.48
Life activities	58.77	53.83	51.51	0.60	0.74
Work and school activities	51.94	42.40	42.94	1.41	0.49
Participation	61.00	67.11	43.93	13.32	0.01*

* , Significant at *p* < 0.05.

SCI, spinal cord injury; TBI, traumatic brain injury.

**TABLE 5:** Relationship between expressed rehabilitation needs (as scored by modified Needs Assessment Questionnaire) and domains of disability (as scored by World Health Organization disability assessment schedule 2.0 (*n* = 105)).

	Cognition		Mobility		Self-care		Getting along with people		Life activities		Work school activities		Participation	
	*r*	*p*	*r*	*p*	*r*	*p*	*r*	*p*	*r*	*p*	*r*	*p*	*r*	*p*
Mobility	0.34	0.01*	0.80	0.01*	0.70	0.01*	0.62	0.01*	0.65	0.01*	0.66	0.01*	0.70	0.01*
Self-care	0.40	0.01*	0.65	0.01*	0.85	0.01*	0.61	0.01*	0.74	0.01*	0.75	0.01*	0.69	0.01*
Home care	0.34	0.01*	0.57	0.01*	0.69	0.01*	0.62	0.01*	0.65	0.01*	0.71	0.01*	0.65	0.01*
Communication	0.36	0.01*	0.58	0.01*	0.74	0.01*	0.57	0.01*	0.63	0.01*	0.67	0.01*	0.61	0.01*
Service in home and community	0.33	0.01*	0.68	0.01*	0.77	0.01*	0.70	0.01*	0.70	0.01*	0.73	0.01*	0.68	0.01*
Employment/work	0.15	0.15	0.33	0.01*	0.54	0.01*	0.51	0.01*	0.51	0.01*	0.58	0.01*	0.37	0.01*
Rehabilitation and medical	0.17	0.08	0.58	0.01*	0.53	0.01*	0.51	0.01*	0.43	0.01*	0.48	0.01*	0.55	0.01*
Other support in the community	0.30	0.01*	0.68	0.01*	0.70	0.01*	0.65	0.01*	0.61	0.01*	0.67	0.01*	0.66	0.01*
Feelingsand memory	0.20	0.04*	0.57	0.01*	0.54	0.01*	0.53	0.01*	0.41	0.01*	0.47	0.01*	0.56	0.01*
Social/recreational	0.09	0.37	0.55	0.01*	0.45	0.01*	0.53	0.01*	0.45	0.01*	0.46	0.01*	0.46	0.01*
Financial/government assistance	0.27	0.01*	0.76	0.01*	0.62	0.01*	0.59	0.01*	0.52	0.01*	0.55	0.01*	0.68	0.01*
Barriers	0.23	0.02*	0.70	0.01*	0.64	0.01*	0.57	0.01*	0.60	0.01*	0.66	0.01*	0.76	0.01*

* , Significant at *p* < 0.05.

mNAQ, modified Needs Assessment Questionnaire; WHODAS, World Health Organization disability assessment schedule.

### Expressed expectations/needs of people with neurological condition from rehabilitation

Four stroke survivors, three patients with SCI and one patient with TBI participated in the FGDs. Participants in the FGDs (5 males, 3 females) were aged 48.10 ± 12.18 years. All the participants agreed that they had needs that are not being specifically addressed by the healthcare system. Needs expressed during the FGDs were categorised into the following themes: physical health need, financial need, health services/rehabilitation need, and emotional and social support need.

#### Physical health need

The greatest need expressed by the participants was the need for restoration of mobility. Participants believed they could continue with some of their former activities and achieve their dreams and aspirations if they were able to move around as illustrated by the following quotes:

‘I need mobility. That is the first thing.’ (P1, SS, male)

‘ehnn …, my first need is mobility … I am believing God to restore me to my formal …’ (P2, SS, female)

‘My present challenge is mobility…the only thing that matters to me now is to walk, … back on my feet … my condition is not acceptable. I can never accept it … I can’t accept this chair (wheel chair).’ (P3, SCI, female)

‘The only thing I need now is my leg.’ (P4, SS, male)

Most of the participants believed that mobility is crucial to enjoying life activities. They perceived mobility as the way to independence:

‘I believe that if I am mobile, that there are a lot of places I can go by myself. My present condition is slowing me down in a lot of things. I do little work and get tired.’ I (P2, SS, female)

‘I have a farm but presently I can’t go to the farm. I can’t afford to pay my workers again. And I can’t go to ask for loan from banks. They will say, ah, you. If you die who will pay. So, the farm is now in the bush. I have 10 hectares. Nobody is ready to assist somebody that is sick.’ (P1, SS, male)

‘I had so many plans like after school. I was supposed to go for youth service but I could not go because I can’t walk. I skipped it till I can walk. I hope to further my education and go for Masters. But due to this situation, I cannot do anything.’ (P3, SCI, female)

#### Financial need

All the participants reported that management of the disabilities resulting from NCs was financially challenging:

‘Financial support is really a serious issue…’ (P5, SCI, male)

‘Financial need is general to everybody especially for those who are not mobile…’ (P6, TBI, male)

‘… Because of poverty in the country. Right from day one, the doctor know that I am supposed to undergo MRI test but because of cost and non-availability of the test (as at that time due to industrial action), they could not inform me till they wasted three years of going to physiotherapy, raise leg, raise hand and doing unnecessary tests uncountable times including X-ray.’ (P3, SCI, female)

‘Yes, even though I need a wheel chair, I will need to restructure my house to aid movement with a wheel chair. I am going to need money to do that.’ (P5, SCI, male)

‘…There are many things…like material needs in terms of appliances, that can assist my arm to be more functional.’ (P1, SS, male)

‘I will need like a motorized wheel chair, it will go a long way to move around without calling for assistance.’ (P3, SCI, female)

Some of the participants reported skipping scheduled physiotherapy appointments because of non-availability of funds:

‘I pay between 1800–2000 naira every week for physiotherapy not to talk of consultant services etc… These costs are too much. It may discourage one from coming for physiotherapy.’ (P6, TBI, male)

‘Like this money thing, sometimes one might not be buoyant enough to come to physio. and you really need the exercises. I don’t know if anything can be done concerning it because I could not attend for like four months because of financial problems.’ (P5, SCI, male)

A participant related the lack of finances to cater for healthcare services to inability to engage in paid employment because of the onset of NC:

‘You know if one is working, to come to hospital you can easily get money to borrow…but as you are now….’ (P7, SS, male)

#### Health services/rehabilitation needs

Participants decried lack of and/or inadequate manpower, especially of trained physiotherapists in healthcare facilities. They believed that there was a disproportion between available physiotherapists and the number of patients/clients on clinic days, which may hinder optimum rehabilitation. This, they opined, constituted a barrier to their rehabilitation need being met.

Participants expressed severe dissatisfaction with physiotherapy services, such as not receiving adequate attention during clinic days, partly because of large number of patients and few attending physiotherapists. They complained about physiotherapists attending to multiple patients at the same time, resulting in sub-optimal therapy sessions. Some participants complained that their clinic appointments were reduced without information on reasons for the reduction. Also, patients claimed physiotherapists did not provide sufficient information on the management of NCs, partly because of consideration of financial capacity of participants. The inadequate provision of information was considered to be problematic:

‘I am not satisfied with Physio services. I am not satisfied with their services. Initially they were seeing me twice a week but they reduced it to once a week. I am so eager to be on my feet because according to my surgeons after surgery I should start walking maybe in 6 months. And this is over 2 years. I am not really satisfied with what I am seeing in my health. I am not seeing any improvement. I am trying to plan my life to go back to school…Since after this surgery it’s been difficult for me as I have pains all over my body. I try as much as possible to smile. When people see me, they think all is well but it is not. I feel unbearable pains. I can’t sleep well at night. I don’t feed well. I wish to eat and if I do, I have indigestion.’ (P3, SCI, female)

‘You see, when you come here, you can see that I am sweating. My singlet is soaked. The AC is not functioning. And for someone who cannot walk, the conveniences are closed. Some people can’t hold their urine and before they go to the other far convenience they end up urinating on themselves.’ (P4, SS, male)

‘Access to physiotherapy services is very important because distance from such services may even be a barrier. So there’s need to make physiotherapy services accessible and available to people who need it.’ (P5, SCI, male)

They also advocated for more physiotherapy clinics and rehabilitation centres:

‘…if we have more functional facilities with sophisticated equipment that can complement…, I believe the pressure will be lesser. If it can’t be within each local government, I think it should be in each senatorial district.’ (P1, SS, male)

‘Probably the hospital is short staffed. When you put a patient on the mat, one PT is attending to 3–4 patients at a time. Raise your leg 10× I am coming. He is going to another person who may be lazy. He might have done it 3× but by the time the PT comes back, he will say he is done. Eventually the goal of treatment is not achieved.’ (P7, SS, male)

Participants advocated for home visits and follow up of patients with disabilities discharged from inpatient care. According to some participants, the social/welfare departments of the healthcare facilities should be proactive in this area. They believed that a monthly follow up of patients after hospital discharge might help in improving their health status.

‘Well I have not seen my doctors (physicians) for over 7 months now…does it mean that the hospital has too much patients that they forget some of their patients. They never checked on me like call this girl or they have forgotten…if only is just to find out…how we are faring, what the situation is like…’ (P3, SCI, female)

#### Emotional/social support needs

Participants expressed the need for more care, love and affection from their family, friends, colleagues and the society at large. According to the participants, a large proportion of the society tend to stigmatise and discriminate against them. Societal attitudes caused discouragement and depression, which participants claimed retards their rate of recovery. Participants expressed the need to dispel the myths surrounding NCs. According to the participants, these myths contributed to the negative societal attitudes towards people with NCs:

‘If you see the way people talk to us and look at us … is it my fault? it’s not my fault o…’ (P3, SCI, female)

‘It’s not as if I don’t have the money to pay for transport. But sometimes when I call them (transporters) they don’t come. They feel going to take this girl again…’ (P8, SCI, female)

‘most of my friends don’t even know about my present condition…also my family. They don’t understand my condition. They feel by now I would have been standing. Sometimes my family reacts to my condition with such statements as ‘we’ve tried o, why are you still sitting? What is still wrong?’ I told them to go and browse about spinal cord injury. At least they will know more about it. At a point my mother understood that recovery is a gradual process not magical.’ (P3, SCI, female)

Some participants decried losing their friends and colleagues because of their NCs. According to participants, these attitudes increased stigmatisation, social isolation and emotional imbalance:

‘I feel so bad seeing myself in this condition. Most of my friends don’t call me again. They don’t want to come close to me again because of the new condition (Spinal cord injury). I feel so bad. I think discrimination is so bad on us with disability…the discrimination is much. When I hear some people say stuff, I feel more pain. And things like this can worsen a person’s condition.’ (P8, SCI, female)

‘…It was my friend that came to ask me if my husband is able to perform his role with me…eh…your husband will be enjoying himself outside o. you better get up on time or else they will snatch your husband and go. I was thinking…’ (P2, SS, female)

One of the participants stated that her inability to walk around has forced her to live a lonely life that limited her interaction with her neighbourhood:

‘Due to my mobility challenge, I don’t go out except on clinic days. I’m always indoors. Sometimes I don’t even go out for a full month. In my estate, nobody knows me. I don’t even know the people I live with. I remember somebody from my estate saw me returning from church and asked if I lived here. He said they have not seen me before…because of that mobility problem.’ (P2, SS, female)

In conclusion, participants suggested government involvement in sensitising and educating the public on the need to provide care and to show empathy towards persons with disabilities as a means of reducing discrimination. Considering the importance of knowledge in handling some of the social needs of people with NCs, participants advocated for educating their family members, friends and the society about NCs:

‘I think advocacy. At least concerned persons should advocate to the government concerning us with disability. The relationship is not good. At least people should show us love. They should know that no condition is ever permanent…we never prayed for it to be so…I just want people to relate more with us than discriminate.’ (P3, SCI, female)

## Discussion

All the individuals with NCs in our study reported unidentified or unmet rehabilitation needs. Their reported needs were in all domains of the mNAQ, which correspond with major areas of independent living. Social and recreational needs were experienced as the most challenging needs by the participants. This is similar to earlier reports amongst individuals with TBI and stroke survivors. Individuals with TBI have been reported to have higher unmet social/recreational needs compared with those without a disability (Brown, Gordon & Spielman 2003). Yi et al. (2015) also reported decreased participation in leisure activity after stroke. According to Mulligan et al. (2012), functional or physiological impairment can make accessing recreational facilities and equipment safely a challenge for persons with NCs. Meanwhile, it has been suggested that providing opportunities for individuals with NCs to participate safely in physical activities within the community could maintain or improve the health status and reduce the need for caregiver assistance (Salbach et al. 2014).

Rehabilitation and medical needs ranked next to social and recreational needs in our study’s participants. Unmet service needs have been reported amongst stroke survivors (Chen et al. 2019). The need for information and more physiotherapy sessions was also highlighted by the participants. This is similar to reports from an earlier study, in which stroke survivors expressed more need for therapies, including physiotherapy (Brandriet, Lyons & Bentley 1994). The need for more physiotherapy sessions have similarly been reported amongst stroke survivors in Nigeria (Olaleye, Hamzat & Akinrinsade 2017). Physiotherapy is considered important for recovery of function amongst stroke survivors, and a decrease in therapy sessions, in terms of frequency and unit time/length, is opined to hinder recovery as expected (Wiles et al. 2002, 2004). Participants also expressed dissatisfaction with physiotherapy services received. The reason for dissatisfaction was mainly a perceived unjust reduction in therapy sessions. This is contrary to previous findings on satisfaction with physiotherapy services amongst Nigerians with chronic diseases (Odebiyi et al. 2009; Olaleye et al. 2017).

A key finding from the FGDs is that nearly all the participants wanted their mobility to be restored. Restoration of mobility was related to walking. This is similar to the report of Aspinal et al. (2014) where walking was regarded as a key indicator of physical health and functioning in people with long-term NCs. In earlier reports by Moreland et al (2009) and Depaul et al. (2013), needs and barriers related to motor control, walking, transfers, wheelchair use, balance and mobility skills were amongst the most expressed needs amongst stroke survivors. Rauch et al. (2011) similarly reported needs relating to participation, mobility, self-care and social integration as the most expressed needs by people with SCI. A mobility limitation is common amongst individuals with NCs (Mulligan et al. 2012). The findings from our study and the previous studies involving people with NCs suggest that mobility is a key priority in rehabilitation for individuals with NCs. Our findings could also be an indication that participants have limited understanding of the motor consequences of NCs or failed to accept that mobility impairments in NCs cannot necessarily be cured and may have to be managed or compensated for.

The accounts of the participants in the FGDs indicated a high need for emotional, social and government support. Participants recounted their experiences that border on maltreatment by friends and members of the community, which led to their isolation from communal and social life. Studies have reported a lack of social support amongst stroke survivors (Lynch et al. 2008; Urimubenshi & Rhoda 2011). Although the desired government support amongst our participants was mostly financial, there included a need for policies to support and protect the rights of people with disabilities within society. Overall, participants with SCI reported the greatest needs in seven domains of the mNAQ more than stroke survivors and individuals with TBI. Studies have reported a higher level of disability in individuals with SCI compared with other populations with disabilities (Aidinoff et al. 2011; Coura et al. 2012; Fernhall et al. 2008). This high level of disability amongst individuals with SCI may contribute to their greater needs.

Needs were negatively correlated with the age of the participants in all but home care, and services at home and community domains on the mNAQ. This suggests a decrease in rehabilitation needs with increasing age. This is similar to the findings of Olaiya et al. (2017) who reported fewer unmet needs with increasing age amongst stroke survivors. A finding of decreasing need with increasing age amongst patients with multiple sclerosis has also been reported (Ponzio et al. 2015). In our environment, people tend to accept morbidity as an inevitable part of aging, and this may account for the low level of needs amongst the elderly.

Female respondents reported higher needs in all domains and barriers to enjoying life compared with male respondents. This is contrary to the findings of Trezzini et al. (2019), where expressed needs were not associated with gender in individuals with SCI despite female participants in their study reporting higher unmet needs for recreation and peer support. Our findings could be a reflection of the demands and expectations placed on women in our environment. The pressure to return to paid employment amongst women who were breadwinners in their family could have caused anxiety and resulted in greater expressed needs.

Participants with secondary education had the greatest needs in all the domains on the mNAQ compared with those with primary education and tertiary education. This finding may be a reflection of the level of exposure of the participants to information. Participants with primary education may have limited information regarding their condition and the possible interventions that are available for amelioration. It could also be that participants with primary education did not express their needs for fear of their inability to pay for services required to meet them. Participants with tertiary education, on the other hand, may be able to take measures to minimise or prevent the negative impact of NCs based on the information available to them. Also, participants with tertiary education could be employed and have health insurance coverage, which could contribute to their needs being met as they arose.

There was a significant linear relationship between needs and severity of disability. This is similar to the findings from the studies of Depaul et al. (2013), Moreland et al. (2009) and Tistad et al. (2012) amongst stroke survivors. According to these authors, stroke survivors with a higher level of disability reported greater rehabilitation needs than those with a lower level of disability. Noreau et al. (2014) and Trezzini et al. (2019) similarly reported that individuals with more severe SCI were more likely to express more needs than those with less severe injury. This suggests that disability is an important driver of rehabilitation needs in people with NCs.

Using a mixed-methods design to investigate the rehabilitation needs of patients with NCs has provided an in-depth understanding of their needs and how and who they feel could meet these needs. Questions in the focus interview guide were derived from items on the mNAQ. Data collected from FGDs complemented the findings from the survey. There are, however, inherent limitations to our study. Participants in the survey were at different stages in their rehabilitation programme. There could be variation in needs based on the stage of rehabilitation, and this could affect the generalisability of findings to all phases of rehabilitation. Both components of our study were based on self-reports from participants, and there could be response bias.

## Conclusion

Rehabilitation needs of individuals with NCs in Nigeria were correlated with severity of disability and differed across NCs. Individuals with SCI, especially those with more severe disability, had greater rehabilitation needs. Lack of mobility was considered a major barrier to enjoying life activities. Participants felt that the provision of transportation for people with disability by the government may minimise barriers to enjoying life. Needs expressed by patients should be considered when planning rehabilitation programmes for people with NCs. Further research on how the needs of patients influence utilisation of rehabilitation services is recommended.

## Data dissemination

The findings from our study underscore the need for rehabilitation therapists to acknowledge and incorporate patients’ needs and expectations in rehabilitation plans. To this end, our findings have been disseminated as platform presentations at the International Conference of Medical Rehabilitation Professionals held at the Lagos Airport Hotel, Lagos, Nigeria from 20 to 22 September, 2017, and at the fifth International Conference on Physiotherapy held in Dubai, UAE from 27 to 29 November, 2017.

## Appendix 1

**TABLE 1-A1:** Good reporting of a mixed-methods study checklist of items.

Item No	Guideline	Where located (Section: page)
1	Describe the justification for using a mixed-methods approach to the research question	Introduction: 4; 43–50
2	Describe the design in terms of the purpose, priority and sequence of methods	Methods: 4; 57–59 Procedure: 7; 134–155
3	Describe each method in terms of sampling, data collection and analysis	Methods: 4–8
4	Describe where integration has occurred, how it has occurred and who has participated in it	4; line 58 7; 134 8; 175 16; 466–468
5	Describe any limitation of one method associated with the present of the other method	16; lines 468–471
6	Describe any insight gained from mixing or integrating methods	16; lines 465–468

*Source*: O’Cathain, A., Murphy, E. & Nicholl, J., 2008, ‘The quality of mixed methods studies in health services research’, *Journal of Health Service Research and Policy* 13(2), 92–98. https://doi.org/10.1258/jhsrp.2007.007074

## Appendix 2

### Focus interview guide for exploring the rehabilitation needs of individuals with neurological conditions

1. Can you please explain some of the treatment you have been receiving because you have this condition?
2. Can you please tell me the things you feel you need from the treatment you are receiving based on your condition?
  Probes: why do you think that is what you need?
  How can providing for this need help or improve your condition?
3. Would you say the care you are receiving cater for the needs you have on account of your condition?
  Probes: How so?
  How not so?
4. What do you think can be performed to meet your needs?
  Probes: how do you feel these things can be performed?
  Who do you think can do these things for you?
5. Is there anything else you would like to tell us?
